# Diversity and community of culturable endophytic fungi from stems and roots of desert halophytes in northwest China

**DOI:** 10.3897/mycokeys.62.38923

**Published:** 2020-02-03

**Authors:** Jia-Long Li, Xiang Sun, Yong Zheng, Peng-Peng Lü, Yong-Long Wang, Liang-Dong Guo

**Affiliations:** 1 State Key Laboratory of Mycology, Institute of Microbiology, Chinese Academy of Sciences, Beijing, 100101, China Institute of Microbiology, Chinese Academy of Sciences Beijing China; 2 National Joint Engineering Research Center of Separation and purification technology of Chinese Ethnic Veterinary Herbs, Tongren Polytechnic College, Tongren, 554300, China University of Chinese Academy of Sciences Beijing China; 3 College of Life Sciences, University of Chinese Academy of Sciences, Beijing, 100049, China Tongren Polytechnic College Tongren China; 4 Department of Molecular Biology and Ecology of Plants, Faculty of Life Sciences, Tel Aviv University, Tel Aviv, 69978, Israel Tel Aviv University Tel-Aviv Israel; 5 School of Geographical Sciences, Fujian Normal University, Fuzhou 350007, China Fujian Normal University Fuzhou China

**Keywords:** community composition, desert halophyte, endophytic fungi, host preference, richness, tissue preference

## Abstract

Halophytes have high species diversity and play important roles in ecosystems. However, endophytic fungi of halophytes in desert ecosystems have been less investigated. In this study, we examined endophytic fungi associated with the stem and root of ten halophytic species colonizing the Gurbantonggut desert. A total of 36 endophytic fungal taxa were obtained, dominated by *Alternaria eichhorniae*, *Monosporascus ibericus*, and Pezizomycotina sp.1. The colonization rate and species richness of endophytic fungi varied in the ten plant species, with higher rates in roots than in stems. The endophytic fungal community composition was significantly affected by plant identity and tissue type. Some endophytic fungi showed significant host and tissue preferences. This finding suggests that host identity and tissue type structure endophytic fungal community in a desert ecosystem.

## Introduction

Endophytic fungi live within plant organs for some time or throughout their life, without causing apparent harm to their host (Petrini 1991). They are widely distributed and significantly contribute to the biodiversity in natural ecosystems (Rodriguez et al. 2009; Porras-Alfaro and Bayman 2011; Hardoim et al. 2015; Yao et al. 2019). These fungi are beneficial to host plants by improving growth performance (Waller et al. 2005; Kannadan and Rudgers 2008; Behie et al. 2012; Khan et al. 2016), providing tolerance against abiotic and biotic stresses (Arnold et al. 2003; Waller et al. 2005; Kannadan and Rudgers 2008; Rodriguez et al. 2008; Hartley and Gange 2009; Yuan et al. 2016). Moreover, endophytic fungi participate in waste decomposition and recycling of nutrients in natural ecosystems (Promputtha et al. 2010; Sun et al. 2011; Purahong et al. 2016). Therefore, understanding the relationship between the endophytic fungal community and host plants is critical to comprehend diversity maintenance and ecosystem function (Hoffman and Arnold 2008; Porras-Alfaro and Bayman 2011; Hardoim et al. 2015).

The endophytic fungal colonization rate, diversity, and community composition is affected by host species, tissue types, and abiotic factors (e.g., Collado et al. 1999; Arnold and Lutzoni 2007; Arfi et al. 2012; Sun et al. 2012a; U’Ren et al. 2012; Lau et al. 2013; Li et al. 2016). For example, Sun et al. (2012a) reported that the host species and tissues types conspicuously affect endophytic fungal community in three woody plants in a mixed temperate forest in China, where the overall colonization rates of endophytic fungi were significantly higher in twigs than in leaves, i.e., twigs harbored more endophytic taxa than leaves. Massimo et al. (2015) suggested that the endophytic fungal community composition in aboveground tissues (branches, stems, and leaves) of Sonoran Desert trees and shrubs were different among host species. However, most previous studies have focused on endophytic fungi of the aerial parts of plants, while very few studies investigated the difference of endophytic fungal community inhabiting the aboveground and belowground plants in ecosystems (Herrera et al. 2010; Márquez et al. 2010; Su et al. 2010; Xing and Guo 2011; Porras-Alfaro et al. 2014). For example, Su et al. (2010) illustrated that *Stipa grandis* inhabited the Inner Mongolia steppe, the colonization rates of endophytic fungi were significantly higher in roots than in leaves, and the endophyte diversity, as well as the composition, was also significantly different in roots or leaves. Recent studies showed the functional importance of endophytic fungi colonized in roots and boosted research interests to root endophytic fungi (Hiruma et al. 2016; Almario et al. 2017; Polme et al. 2018; Schroeter et al. 2019). The difference in the endophytic fungal community among the aboveground and belowground of harsh habitat plants is an important scientific question.

Halophytes constitute about 1% of the world’s flora, survive and reproduce in saline habitats such as coastal and salinized inland regions (Flowers et al. 1986; Flowers and Colmer 2008; Ward 2009). These halophytes contain grasses, shrubs, and trees, which constitute important eco-functional vegetation in the desert and coastal areas (Rozema and Flowers 2008; Chen et al. 2009; Giri et al. 2011). In China, there are 3.69×10^7^ ha of saline soil regions and 555 halophyte species, accounting for 21.3% of the halophytes in the world (Zhao et al. 2013a). Particularly in arid and semiarid northwest China, saline lands are distributed in the Gobi Desert, which accounts for 69% of the total saline lands and accommodates more than 60% of the halophyte resources of China (Zhao et al. 2013b). Halophytes in the desert areas are exposed to multiple environmental stresses, such as low water availability, high salinity, and nutrient deprivation (Ward 2009; Liu et al. 2013), and thus are unique niches for endophytes affected by the harsh environment. However, studies of endophytes in saline environments of China focused on mangroves parallel to the coast (Xing et al. 2011; Xing and Guo 2011; Liu et al. 2012; Li et al. 2016).

Inland halophytes form extensive symbiotic relations with endophytic fungi in harsh environments, which benefit their hosts by promoting resistance against high salinity stress (Rodriguez et al. 2008; Massimo et al. 2015; Khan et al. 2016). A few studies focused on endophytes of halophytes living inland (e.g., Sun et al. 2012b; Macia-Vicente et al. 2012). There are even fewer studies of endophytic fungi that have been carried out on desert halophytes, and they merely focused on endophytes on roots (Sonjak et al. 2009; Macia-Vicente et al. 2012). Moreover, Sun et al. (2012b) aimed at the endophytic fungal community in stems and leaves of desert halophytes in Tennger Desert region of China. Therefore, further study is required on endophytes of halophytes in the desert region to reveal the community of endophytic fungi under arid and salinity stress, with an emphasis on aboveground and belowground parts of plants.

In order to improve our understanding of the endophytic fungi of desert halophytes, we selected ten halophyte species in the Gurbantonggut desert, Xinjiang, northwest China. The endophytic fungi were isolated from the stems and roots of halophytes and identified according to morphological characteristics and molecular data. This study aimed to reveal how the colonization rate, diversity, and community composition of endophytic fungi differed among halophytes species and tissue types. Besides, it will also provide preliminary data of halophyte endophytes for future studies in bioactive natural products, ecosystem reconstruction, or agricultural application in desert regions.

## Methods

### Study site and sampling procedure

The study was carried out at the Fukang Desert Ecosystem Observation and Experiment Station, Chinese Academy of Sciences, located in the southern edge of the Gurbantonggut desert in China (44°17'N–44°22'N, 87°55'E–87°56'E, 448–461 m above sea level). The site has a continental arid temperate climate, with an annual mean temperature of 6.6 °C (a maximum of 44.2 °C in hot, dry summer and a minimum of -42.2 °C in freezing winter) (Dai et al. 2015). The annual mean precipitation is about 160 mm with annual pan evaporation of 2000 mm, resulting in soil with high salinity (0.45–2.25%) (Xu et al. 2007).

On 30^th^ July 2015, we selected ten halophyte species *Bassia dasyphylla* (Fisch. et C. A. Mey.) Kuntze, *Ceratocarpus arenarius* L., *Kalidium foliatum* (Pall.) Moq., *Salsola nitraria* Pall., *Suaeda acuminata* (C. A. Mey.) Moq., *Su. salsa* (L.) Pall. (Chenopodiaceae), *Eragrostis minor* Host (Poaceae), *Reaumuria songarica* (Pall.) Maxim. (Tamaricaceae), *Seriphidium santolinum* (Schrenk) Poljak (Asteraceae), and *Peganum harmala* (L.) (Zygophyllaceae) at the site. Ten healthy individuals of each plant species were uprooted to collect twig and root samples at the location. All sampled individuals of the same species were more than 50 m away from each other, in order to reduce the spatial autocorrelation and recover representative local endophyte community (Li et al. 2016, Yao et al. 2019). The collected samples were immediately placed in autoclaved paper bags, labeled, and transported to the laboratory in an ice-box. Samples were stored at 4 °C and processed within 4 days.

### Isolation and identification of endophytic fungi

Since most of the plant species involved in the current study (except for *E. minor*) possess reduced leaves, which are hard to discern from the stems, we selected only stems to isolate endophytes colonized aerial parts of the plants. Roots and stems of individual plants were cut into 5 mm long segments (ca. 2 mm in diameter). Eight root segments and 8 stem segments were randomly selected from each sample. In total, 1600 segments (10 plant species × 10 individuals × 2 tissue types × 8 segments) were used for endophyte isolation in this study.

Surface sterilization was conducted according to Guo et al. (2000). Segments were surface sterilized by consecutive immersion for 1 min in 75% ethanol, 3 min in 3.25% sodium hypochlorite, and 30 sec in 75% ethanol. Sets of four segments were then evenly placed in a 90 mm Petri dish containing potato dextrose agar (PDA, 2%). Benzylpenicillin sodium (50 mg/L, North China Pharmaceutical Group Corporation, China) was added to suppress bacterial growth. Petri dishes were sealed, incubated for 2 months at 25 °C, and examined periodically. When fungal colonies developed, they were transferred to a new PDA containing Petri dishes for purification. The purified strains were transferred to PDA slants for further study.

Subcultures on PDA were examined periodically, and the sporulated isolates were identified based on their morphological characteristics. The non-sporulated cultures were designated as *mycelia sterilia*, which were divided into different “morphotypes” according to colony color, texture, and growth rate on PDA (Guo et al. 2000). One representative strain of each morphotype or sporulated strain was selected for further molecular identification. The living cultures are deposited in China General Microbiological Culture Collection Center (CGMCC) in Beijing, China.

### DNA extraction, amplification, sequencing, and identification

Genomic DNA was extracted from fresh cultures following the protocol of Guo et al. (2000). Fresh fungal mycelia (ca. 50 mg) were scraped from the surface of the PDA plate and transferred into a 1.5 mL microcentrifuge tube with 700 µL of preheated (65 °C) 2 × CTAB extraction buffer (2% CTAB, 100 mM Tris-HCl, 1.4 M NaCl, 20 mM EDTA, pH 8.0), and 0.2 g of sterilized quartz sand. The mycelium was ground using a glass pestle and then incubated in a 65 °C water bath for 30 min with occasional gentle swirling. Five hundred microliters of phenol:chloroform (1:1) were added into each tube and mixed thoroughly to form an emulsion. The mixture was spun at 12,000 g for 15 min at room temperature in a microcentrifuge, and the aqueous phase was transferred into a fresh 1.5 mL tube. The aqueous phase containing DNA was re-extracted with chloroform:isoamyl (24:1) until no interface was visible. Thirty microliters of 5 M KOAc was added into the aqueous phase followed by 200 µL of isopropanol and inverted gently to mix. The genomic DNA was precipitated at 9200 g for 2 min in a microcentrifuge at 4 °C. The DNA pellet was washed twice with 70% ethanol and dried using SpeedVac (AES 1010, Savant, Holbrook, NY, USA) for 10 min or until dry. The DNA pellet was then re-suspended in 65 µL ultrapure sterilized water.

The internal transcribed spacer (ITS) region of rDNA was amplified using primer pairs ITS4 (White et al. 1990) and ITS1F (Gardes and Bruns 1993). Amplification was performed in a 50 µL reaction volume which contained PCR buffer (20 mM KCl, 10 mM (NH_4_)_2_SO_4_, 2 mM MgCl_2_, 20 mM Tris-HCl, pH 8.4), 200 µm of each deoxyribonucleotide triphosphate, 15 pmols of each primer, 100 ng template DNA, and 2.5 U *Taq* polymerase (Biocolor BioScience & Technology Company, Shanghai, China). The thermal cycling program was as follows: 3 min initial denaturation at 94 °C, followed by 35 cycles of 30-sec denaturation at 94 °C, 30-sec annealing at 52 °C, 1 min extension at 72 °C; and a final 10 min extension at 72 °C. A negative control using water instead of template DNA was included in the amplification process. Four microliters of PCR product from each PCR reaction were examined by electrophoresis at 80 V for 30 min in a 1% (w/v) agarose gel in 1 × TAE buffer (0.4 M Tris, 50 mM NaOAc, 10 mM EDTA, pH 7.8) and visualized under ultraviolet (UV) light after staining with ethidium bromide (0.5 µg/mL). PCR products were purified using Wizard SV Gel and PCR Clean-Up System (Promega, Madison, USA) and directly sequenced with primer pairs, as mentioned above in the ABI 3730-XL DNA sequencer (Applied Biosystems, Inc. USA).

A value of 97% of ITS region identity was used as a DNA barcoding threshold for OTU clustering (O’Brien et al. 2005). The taxonomical assignments for each OTU were determined according to the BLAST results against both UNITE+INSD (UNITE combined international nucleotide sequence databases) and GenBank public sequence databases. A representative sequence of each OTU was selected and searched against the UNITE+INSD fungal ITS databases (Kõljalg et al. 2013) using a basic local alignment search tool (BLAST) (Altschul et al. 1990). The DOIs of UNITE fungal Species Hypothese at 1.5% threshold (Nilsson et al. 2019) were also added to each of taxonomical assigments (Table 1). For reliable identification of the fungi, a representative sequence of each OTU was searched against the GenBank public sequence databases using BLASTN (Sun et al. 2011, Sun et al. 2012a). For further identification of these fungi, we select the most reliable sequence as a reference (the sequences originated from mycologists or taxonomists, yielded from taxonomical or phylogenetical studies, or were part of cultures or speciemens in famous collections, would be given higher credits). As for taxonomical levels higher than species, we typically relied on 90, 85, 80, and 75% sequence identity as a criterion for assigning OTUs with names of a genus, family, order, or class, respectively (Tedersoo et al. 2014). Nevertheless, the results of sequence-based identification were calibrated with morphological characteristics in our study given the strains within one OTU sporulated. The microscopic observation was applied with cultures mounted in sterile water using a compound microscope (Zeiss Axio Imager A2, Carl Zeiss Microscopy, Göttingen, Germany). The ITS sequences of endophytic fungi obtained in this study have been deposited in National Center for Biotechnology Information (NCBI) with GenBank accession no. KY114893 to KY114928 (Table 1).

**Table 1.** Molecular identification of endophytic fungi based on ITS sequences.

Fungal taxa	accession no.	Closest blast match in GenBank (accession no.)	Identity (%)	UNITE taxon name (SH code at 1.5% threshold)
*Acremonium alternatum*	KY114893	*Acremonium alternatum* (AY566992)	100	Pezizomycotina (SH1560626.08FU)
*Alternaria chlamydospora*	KY114895	*Alternaria chlamydospora* (NR136039)	99	*Alternaria chlamydospora* (SH1505867.08FU)
*Alternaria eichhorniae*	KY114894	*Alternaria burnsii* (KR604836)	100	*Alternaria eichhorniae* (SH1526398.08FU)
*Aspergillus flavus*	KY114898	*Aspergillus flavus* (KU296258)	100	*Aspergillus flavus* (SH1532605.08FU)
*Aspergillus fumigatiaffinis*	KY114899	*Aspergillus fumigatiaffinis* (MH474422)	100	*Aspergillus fumigatus* (SH1529985.08FU)
*Aspergillus terreus*	KY114900	*Aspergillus terreus* (KM249873)	100	*Aspergillus terreus* (SH1530841.08FU)
*Aureobasidium pullulans*	KY114901	*Aureobasidium pullulans* (MH857648)	100	*Aureobasidium pullulans* (SH1515060.08FU)
*Bipolaris prieskaensis*	KY114902	*Bipolaris prieskaensis* (JQ517482)	100	*Bipolaris prieskaensis* (SH1526609.08FU)
*Cladosporium limoniforme*	KY114903	*Cladosporium limoniforme* (KT600401)	100	*Mycosphaerella tassiana* (SH1572792.08FU)
*Curvularia inaequalis*	KY114905	*Curvularia inaequalis* (KT192305)	99	*Curvularia inaequalis* (SH1526407.08FU)
*Didymella glomerata*	KY114906	*Didymella glomerata* (FJ427004)	99	*Didymella exigua* (SH1547057.08FU)
*Fusarium avenaceum*	KY114907	*Fusarium avenaceum* (JN631748)	100	*Gibberella tricincta* (SH1546323.08FU)
*Fusarium incarnatum*	KY114908	*Fusarium incarnatum* (KT748520)	100	*Gibberella intricans* (SH1610158.08FU)
*Fusarium oxysporum*	KY114909	*Fusarium oxysporum* (EU429440)	100	*Gibberella fujikuroi* (SH1610157.08FU)
*Fusarium proliferatum*	KY114910	*Fusarium proliferatum* (KP132229)	100	*Fusarium proliferatum* (SH1610159.08FU)
*Humicola fuscoatra*	KY114911	*Humicola fuscoatra* (KP101183)	99	Pezizomycotina (SH1642162.08FU)
*Monosporascus ibericus*	KY114912	*Monosporascus ibericus* (JQ973832)	97	*Monosporascus ibericus* (SH1578625.08FU)
*Monosporascus* sp.	KY114913	*Monosporascus* sp. (KT269082)	97	*Monosporascus* (SH1578615.08FU)
*Neocamarosporium obiones*	KY114896	*Ascochyta obiones* (GU230752)	100	Pleosporales (SH1524225.08FU)
*Neocamarosporium* sp.1	KY114914	*Neocamarosporium goegapense* (KJ869163)	94	*Neocamarosporium salsolae* (SH1524232.08FU)
*Neocamarosporium* sp.2	KY114916	*Neocamarosporium* sp. (KY940767)	97	*Neocamarosporium* (SH1524244.08FU)
*Neocamarosporium* sp.3	KY114897	*Pleospora calvescens* (MH861148)	96	Pleosporales (SH1524225.08FU)
*Neodidymelliopsis polemonii*	KY114915	*Neodidymelliopsis polemonii* (KT389532)	100	*Didymella exigua* (SH1547057.08FU)
*Paraphaeosphaeria sporulosa*	KY114904	*Coniothyrium sporulosum* (DQ865113)	97	*Paraphaeosphaeria sporulosa* (SH1582449.08FU)
Pezizomycotina sp.1	KY114922	*Pleomonodictys descalsii* (NR_154369)	88	Pezizomycotina (SH1574559.08FU)
Pezizomycotina sp.2	KY114923	*Trematosphaeria grisea* (NR132039)	86	Pezizomycotina (SH1574559.08FU)
Pleosporales sp.	KY114917	Pleosporales sp. (KF887149)	96	Pleosporales (SH1582443.08FU)
*Preussia* sp.1	KY114918	*Preussia terricola* (GQ203765)	92	*Preussia terricola* (SH1642175.08FU)
*Preussia* sp.2	KY114919	*Preussia* sp. (HM007080)	99	*Preussia* (SH1541731.08FU)
*Sarocladium kiliense*	KY114920	*Sarocladium kiliense* (KM231849)	99	*Sarocladium kiliense* (SH1541920.08FU)
*Simplicillium obclavatum*	KY114921	*Simplicillium obclavatum* (AB604000)	99	*Simplicillium obclavatum* (SH1584064.08FU)
Trematosphaeriaceae sp.	KY114924	*Medicopsis romeroi* (KF015657)	88	*Medicopsis romeroi* (SH1613813.08FU)
Trichocomaceae sp.	KY114925	*Talaromyces purpureogenus* (KM086709)	86	*Talaromyces marneffei* (SH1516144.08FU)
*Ulocladium oblongo-obovoideum*	KY114926	*Ulocladium oblongo-obovoideum* (MH863976)	100	*Alternaria eichhorniae* (SH1526398.08FU)
*Xylaria hypoxylon*	KY114927	*Xylaria hypoxylon* (KF306342)	100	Xylariaceae (SH1541119.08FU)
Xylariales sp.	KY114928	Xylariales sp. (KC460867)	98	Xylariales (SH1578643.08FU)

### Data analysis

All statistical analyses were carried out in R 3.3.1 (R Development Core Team 2016). The colonization rate of endophytic fungi was calculated as the total number of tissue segments infected by fungi divided by the total number of tissue segments incubated (Sun et al. 2011). The relative abundance was calculated as the number of isolates of a taxon divided by the total number of isolates of all taxa, and the fungal richness was defined as the number of fungal species in a sample.

One-way analysis of variance (ANOVA) was carried out to test the effect of plant species or tissue type (stem and root) on the colonization rate and species richness of endophytic fungi. Multiple comparisons were performed using *post hoc* Tukey’s HSD (Honest Significant Difference) tests to examine the significant differences among the plant species or tissue types at *P* < 0.05 level. All data were tested for normality and homogeneity of variance before ANOVA. In cases where satisfactory results of homogeneity of variance amongst plant species after square root and transformation were not observed (e.g., in stems), then nonparametric Kruskal-Wallis test followed by pairwise comparisons was applied to examine the significant difference among plant species at *P* < 0.05 level. *T*-test was applied to examine the significant difference of the colonization rate and species richness of endophytic fungi between stems and roots for each plant species at *P* < 0.05 level. Canonical correspondence analysis (CCA) was performed to observe the correlation between endophytic fungi and plant species or tissue types with the ‘cca’ function in the vegan package (Oksanen et al. 2019). The effects of plant species and tissue type on community composition of endophytic fungi were tested by permutational multivariate analysis of variance (PermANOVA) using the ‘adonis’ command in the vegan package (Oksanen et al. 2019).

The host-fungus association preferences were evaluated based on a *d*’ interaction specialization index (Blüthgen et al. 2007) using the ‘dfun’ function in the bipartite package (Dormann et al. 2009) according to Toju et al. (2016). Briefly, a binarized sample × fungal taxon matrix (i.e., presence/absence) was converted into a ‘species-level’ matrix, in which rows depicted plant species, columns represented endophytic fungal taxa, and cell entries were the number of samples from which respective combinations of plants and fungi were observed. To perform a randomization analysis of the *d*’ index, plant species labels in the sample × fungal taxon matrix were shuffled, and then, the randomized species-level matrices were obtained (1000 permutations). The *d*’ value of each plant species or each fungal taxon was standardized as follows: standardized *d*’ = [*d*’_observed_ - Mean (*d*’_randomized_)] /SD (*d*’_randomized_), where the *d*’_observed_ was the *d*’ estimate of the original data, and Mean (*d*’_randomized_) and SD (*d*’_randomized_) were the mean and standard deviation of the *d*’ scores of randomized data matrices. Also, we evaluated the observed frequency (counts) of each plant-fungus association in the species-level matrix, and quantified with the two-dimensional preferences (2*DP*) in a pair of a plant species (*i*) and a fungal taxon (*j*) based on the species-level original and randomized matrices used in the *d*’ analysis: 2*DP* (*i*, *j*) = [N_observed_ (*i*, *j*) - Mean (N_randomized_ (*i*, *j*))] /SD (N_randomized_ (*i*, *j*)), where N_observed_ (*i*, *j*) denoted the number of samples from which a focal combination of a plant and a fungus was observed in the original data, and the Mean (N_ranodomized_ (*i*, *j*)) and SD (N_randomized_ (*i*, *j*)) were the mean and standard deviation of the number of samples for the focal plant-fungus pair across randomized matrices. The *P* values were adjusted based on the false discovery rate (FDR) (Benjamini and Hochberg 1995).

## Results

### Colonization rate of endophytic fungi

**Figure 1.**
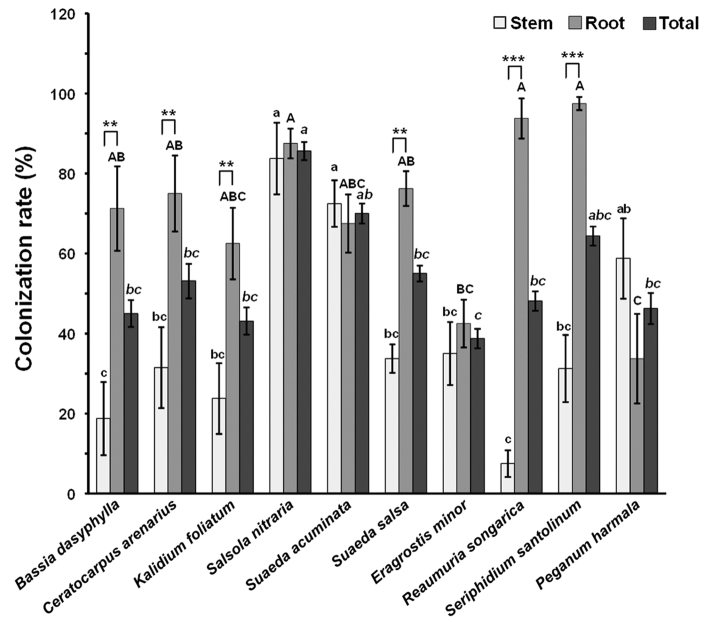
Colonization rate of endophytic fungi in stem, root, and total (stem + root) tissues of the ten halophyte species. Data are means ± SE (n = 10). Columns without shared lowercase, uppercase, and italic letters denote the significant difference in the stem, root, and total tissues among the halophyte species, respectively. Asterisks above bars indicate significant difference between stem and root tissues for each plant species (** *P* < 0.01, *** *P* < 0.001).

A total of 1046 fungal strains were recovered from 1600 tissue segments from ten halophyte species. The colonization rate of endophytic fungi ranged from 7.5 ± 3.33% to 83.75 ± 8.95% in stems, from 33.75 ± 11.19% to 97.5 ± 1.67% in roots, and from 38.75 ± 2.46% to 85.63 ± 2.28% overall for the entire plant among the ten halophyte species (Fig. 1). One-way ANOVA showed that the colonization rate of endophytic fungi was significantly affected by plant identity (*F* = 5.847, *P* < 0.001) and tissue type (*F* = 8.184, *P* < 0.001). In the entire plant, the colonization rate of endophytic fungi was significantly higher in *Sa. nitraria* than in other plants (except for *Su. acuminata* and *Se. santolinum*) and was significantly higher in *Su. acuminata* than in *E. minor* (Fig. 1). In the stem, the colonization rate of endophytic fungi was significantly higher in *Sa. nitraria* and *Su. acuminata* than in the other halophyte species (except for *P. harmala*). For *P. harmala*, the colonization rate of endophytic fungi was significantly higher than in *B. dasyphylla* and *R. songarica* (Fig. 1). In the root, the colonization rate of endophytic fungi was significantly higher in *Se. santolinum*, *R. songarica*, and *Sa. nitraria* than in *E. minor* and *P. harmala*, and was significantly higher in *B. dasyphylla*, *C. arenarius*, and *Su. salsa* than in *P. harmala* (Fig. 1). Furthermore, the colonization rate of endophytic fungi was significantly higher in roots than in stems in *B. dasyphylla*, *C. arenarius*, *K. foliatum*, *Su. salsa*, *R. songarica*, and *Se. santolinum*, but no significant difference was observed in the other four halophyte species (Fig. 1).

### Endophytic fungal richness

**Figure 2.**
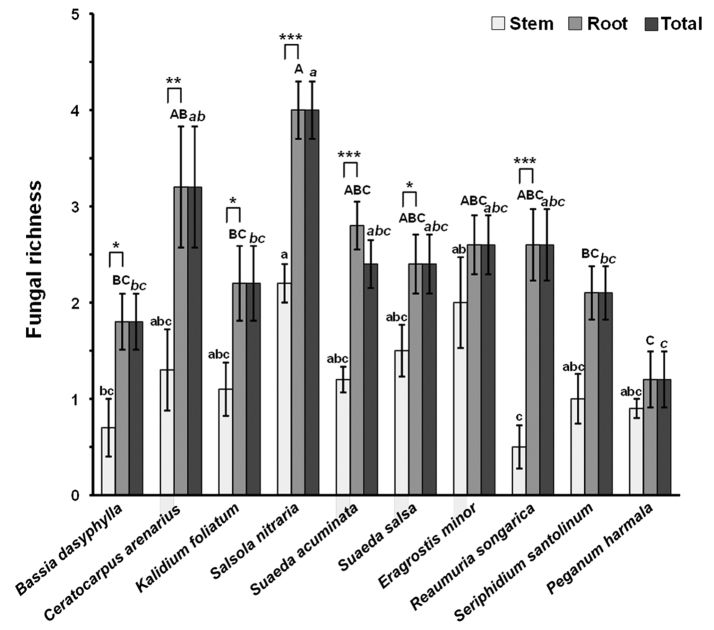
Endophytic fungal richness in stem, root and total (stem + root) tissues of the ten halophyte species. Data are means ± SE (n = 10). Columns without shared lowercase, uppercase, and italic letters denote significant difference in the stem, root, and total tissues among the plant species, respectively. Asterisks above bars indicate the significant difference between stem and root tissues for each halophyte species (* *P*<0.05, ** *P* < 0.01, *** *P* < 0.001).

In total, 36 fungal taxa were isolated and identified based on morphological characters and ITS sequences (Table 1). The richness of endophytic fungi ranged from 0.5 ± 0.22 to 2.2 ± 0.2 in stems, from 1.2 ± 0.29 to 4 ± 0.3 in roots and from 1.2 ± 0.29 to 4 ± 0.3 (means ± SE) in overall among the ten halophyte species (Fig. 2). The richness of endophytic fungi was significantly affected by plant species (ANOVA, *F* = 4.635, *P* < 0.001) and tissue type (Kruskal-Wallis test, *x*^2^ = 34.993, *P* < 0.001). In the stem, the fungal richness was significantly higher in *Sa. nitraria* than in *B. dasyphylla* and *R. songarica*, and significantly higher in *E. minor* than in *R. songarica* (Fig. 2). In the root, the fungal richness was significantly higher in *Sa. nitraria* than in *B. dasyphylla*, *K. foliatum*, *Se. santolinum* and *P. harmala*, and significantly higher in *C. arenarius* than in *P. harmala* (Fig. 2). Furthermore, the fungal richness was significantly higher in roots than in stems in ten plant species, except for *E. minor*, *Se. santolinum* and *P. harmala* (Fig. 2).

### Endophytic fungal community composition

**Figure 3.**
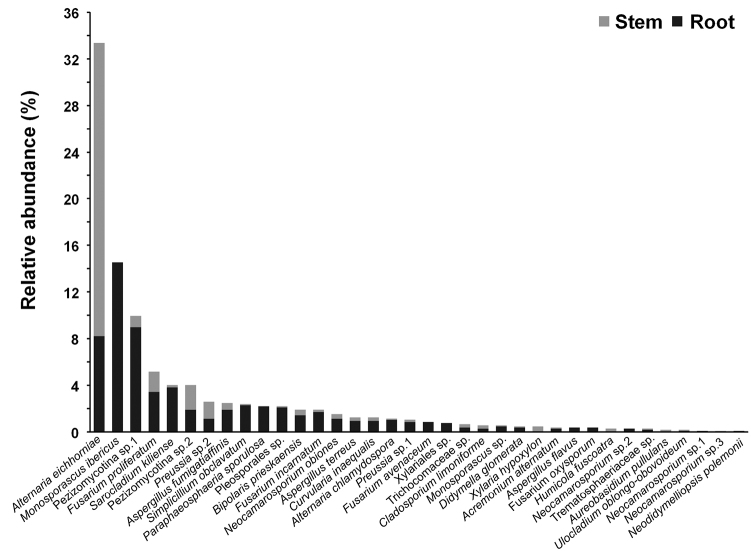
Relative abundance of endophytic fungi in the stem and root tissues of the ten halophyte species.

**Figure 4.**
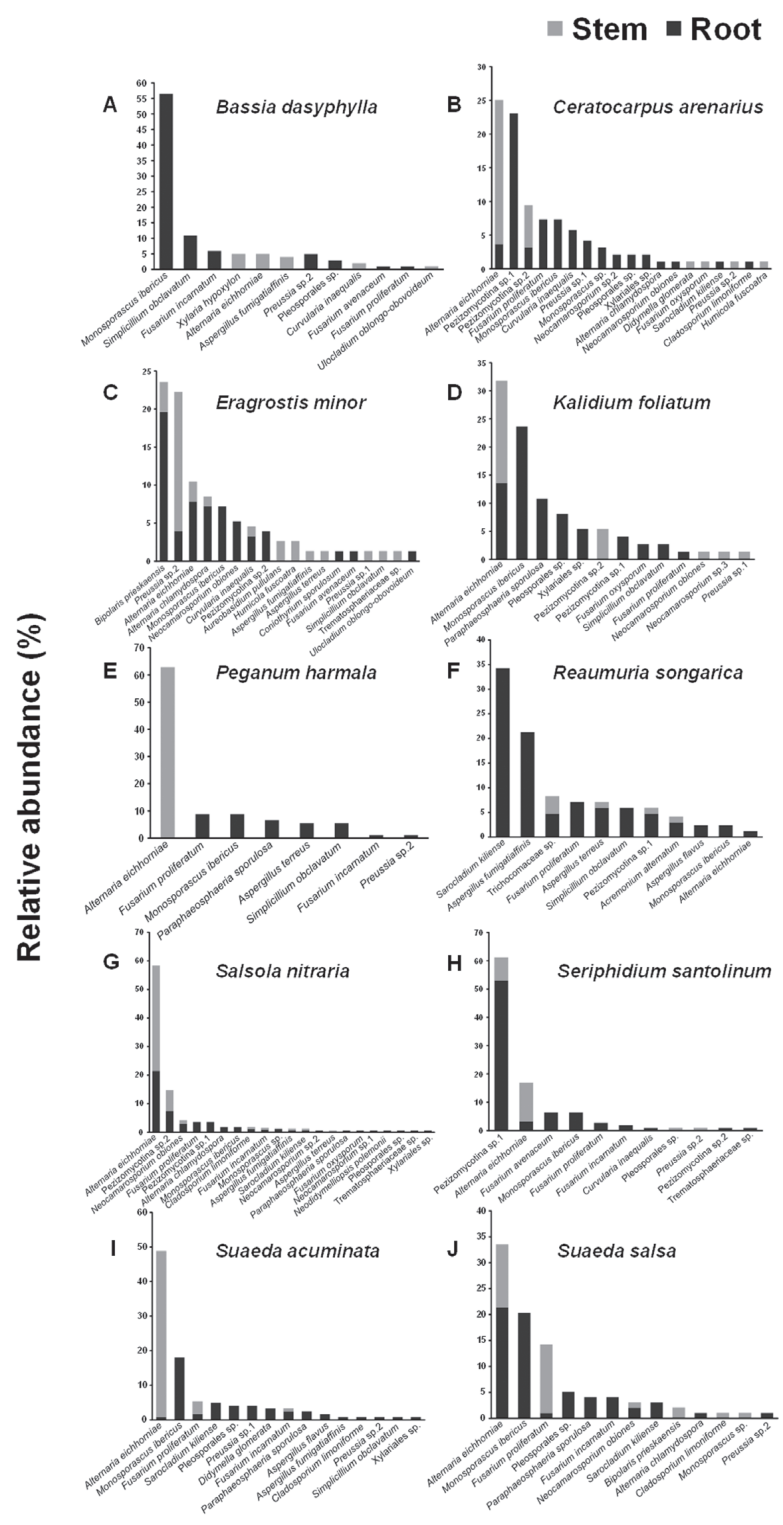
Relative abundance of endophytic fungi in the stem and root of different halophyte species.

Of the 36 endophytic fungi, 32 were recovered from roots, 27 from stems, and 23 were common in both roots and stems (Fig. 3). Among seven abundant endophytic fungi (relative abundance > 15% in certain plant species), *Alternaria eichhorniae* was the most abundant stem endophyte and was recovered from *C. arenarius*, *K. foliatum*, *P. harmala*, *Sa. nitraria*, *Su. acuminata*, and *Su. salsa* (Fig. 4B, D, E, G, I, J). In addition, *Monosporascus ibericus* was exclusively recovered from roots of *B. dasyphylla*, *K. foliatum*, *Su. acuminata*, and *Su. salsa* (Fig. 4A, D, I, J). In *C. arenarius*, Pezizomycotina sp.1 was exclusively recovered from roots, and in *Se. santolinum*Pezizomycotina sp.1 was mostly recovered from roots (Fig. 4B, H). *Sarocladium kiliense* and *Aspergillus fumigatiaffinis* were only found on the roots of *R. songarica* (Fig. 4F). *Bipolaris prieskaensis* was mostly isolated from roots, and *Preussia* sp.2 was mostly distributed in stems in *E. minor* (Fig. 4C).

**Figure 5.**
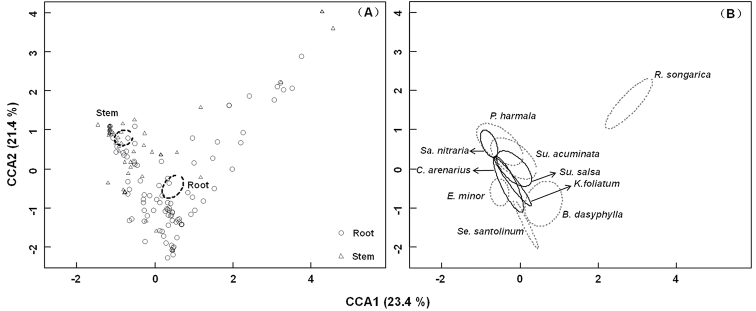
Canonical correspondence analysis (CCA) ordination plot of endophytic fungal communities of stem and root tissues (**A**) and halophyte species (**B**). Dotted ellipses indicate 95% confidence intervals around centroids of tissue type (**A**) and plant species (**B**), *B. dasyphylla* = *Bassia dasyphylla*, *C. arenarius* = *Ceratocarpus arenarius*, *K. foliatum* = *Kalidium foliatum*, *Sa. nitraria* = *Salsola nitraria*, *Su. acuminata* = *Suaeda acuminata*, *Su. salsa* = *Suaeda salsa*, *E. minor* = *Eragrostis minor*, *R. songarica* = *Reaumuria songarica*, *Se. santolinum* = *Seriphidium santolinum*, and *P. harmala* = *Peganum harmala*.

The CCA results indicated that the endophytic fungal community composition was significantly different between stems and roots of the ten halophyte species (Fig. 5A), and significantly different among some plants, such as *R. songarica*, *B. dasyphylla*, *P. harmala* and *E. minor* (Fig. 5B). The PermANOVA results also showed that the endophytic fungal community composition was significantly affected by tissue type (*R*^2^ = 0.212, *P* = 0.001) and plant species (*R*^2^ = 0.082, *P* = 0.001).

### Host-fungus association preferences

**Figure 6.**
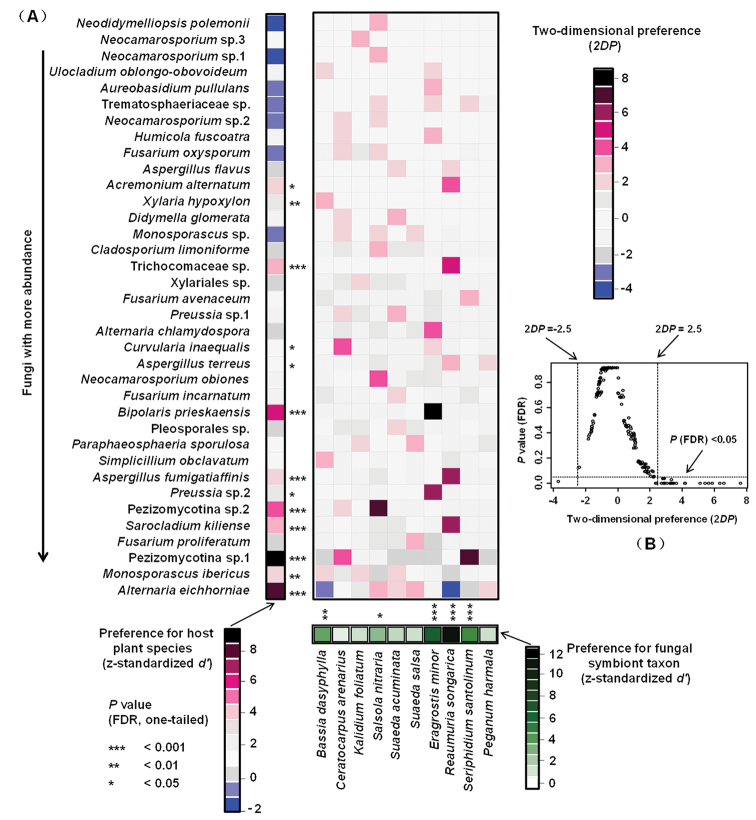
Preferences observed in the plant-fungus associations. **A** Preference scores. The standardized *d*’ estimate of preferences for fungal taxon is shown for each halophyte (column), and the standardized *d*’ estimate of preferences for plant species is indicated for each of the fungal taxon (row). Each cell in the matrix indicates a two-dimensional preference (2*DP*) estimate, which measures to what extent the association of a focal plant-fungus pair was observed more/less frequently than expected by chance. *P* values were shown as false discovery rates (FDRs) in the plant/fungus analysis. **B** Relationship between 2*DP* and FDR-adjusted *P* values, 2*DP* values larger than 2.5 and those smaller than -2.5 represented strong preference and avoidance, respectively (*P*_FDR_ < 0.05). Significance: *, *P* < 0.05, **, *P* < 0.01, ***, *P* < 0.001.

Host-fungus association preference analysis showed that five out of ten halophyte species showed significant preferences to endophytic fungi, especially strong preferences in *E. minor*, *R. songarica*, and *Se. santolinum* (Fig. 6A). Among the 36 endophytic fungi, 13 showed significant preferences for host species, particularly strong preferences were observed in *Al. eichhorniae*, *M. ibericus*, Pezizomycotina sp.1, *Sr. kiliense*, Pezizomycotina sp.2, *As. fumigatiaffinis*, *B. prieskaensis*, Trichocomaceae sp., and *Xylaria hypoxylon* (Fig. 6A). Furthermore, 26 out of 208 pairs of plants and fungi showed significant preferences, such as pairs Pezizomycotina sp.1 and *Se. santolinum*, *Sr. kiliense* and *R. songarica*, *B. prieskaensis* and *E. minor*, *As. fumigatiaffinis* and *R. songarica* (Fig. 6).

## Discussion

The colonization rate and species richness of endophytic fungi varied among desert halophyte species in the current study. Similar results have been reported in previous studies in mangrove (Xing et al. 2011; Xing and Guo 2011; Liu et al. 2012; Li et al. 2016), desert halophytes (Sun et al. 2012b), gypsophilous plants (Porras-Alfaro et al. 2014), desert trees and shrubs (Massimo et al. 2015), and plants in other ecosystem (Su et al. 2010; Sun et al. 2012a). For example, Xing et al. (2011) recovered 39 distinct endophytic species in five mangrove species and found the colonization rate of endophytic fungi ranging from 12.5 to 41.7% in roots, from 8.0 to 54.0% in stems, and from 12.5 to 25.1% in leaves. Sun et al. (2012b) identified 21 endophyte species from eight desert halophytes and found the colonization rates ranging from 35 to 100% in stems and leaves. Furthermore, we found that the colonization rate and species richness of endophytic fungi were generally higher in roots than in stems, which is in contrast with studies carried out in *Holcus lanatus* (Márquez et al. 2010), *Stipa grandis* (Su et al. 2010), and gypsophilous plants (Porras-Alfaro et al. 2014) in arid ecosystem. The difference between endophytic colonization and diversity between above- and below-ground might be attributed to both biotic and abiotic factors. In the study site, humidity is much lower in the air than in the soil, which might result in lower colonization rate and species richness of endophytic fungi in stems than in roots, as endophyte colonization is positively correlated with humidity (Herrera et al. 2010; Massimo et al. 2015). Also, the relatively moist and organic-rich soil substrate is capable of supporting diverse and abundant fungal propagules for penetration in plant roots in comparison to stems (Bridge and Spooner 2001; Massimo et al. 2015).

We found that the endophytic fungi community composition is halophyte species-dependent. Similar results have been reported in some previous studies on halophytes and desert plants (Xing et al. 2011; Xing and Guo 2011; Macia-Vicente et al. 2012; Sun et al. 2012b; Porras-Alfaro et al. 2014; Massimo et al. 2015; Li et al. 2016). For example, Sun et al. (2012b) indicated that the endophytic fungal community in stems and leaves of eight desert plants were different among host species in the Tennger Desert region, China. Massimo et al. (2015) suggested the fungal endophyte community composition differed among host species in the aboveground tissues of Sonoran Desert plants. It has been reported that the host species is a key factor shaping endophyte community structures (Hoffman and Arnold 2008; Arfi et al. 2012; Sun et al. 2012a; Hardoim et al. 2015). Our host preference analysis indicated that 13 endophytic fungal species show significant host preferences. For example, *B. prieskaensis* preferred colonizing *E. minor*, and *Sa. kiliense* and *As. fumigatiaffinis* preferred *R. songarica*. Hardoim et al. (2015) suggested that the selective forces do not act merely on the plant genome itself, but on its associated microbial community also. Moreover, the endophytic fungal composition could be affected by the expected difference in plant chemistry (Arnold et al. 2001; Huang et al. 2008). For example, in the present study, *P. harmala*, a medical plant possessing antifungal properties (Hashem 2011), might inhibit fungal colonization and thus contained the less diverse endophyte community. Therefore, the chemical or physiological traits of plants also affect the endophyte community.

Community composition of endophytic fungi was also affected by plant tissue types (root and stem), which corroborate earlier studies carried out in semi-arid and arid ecosystems (Su et al. 2010; Porras-Alfaro et al. 2014), and highlighted in the review by Hardoim et al. (2015). Despite the dissimilarity in the availability of fungal inocula between above- and under-ground circumstances discussed previously, previous studies suggested that the morphology and chemical substance of tissues also influenced the community composition of roots and stems (Herrera et al. 2010; Su et al. 2010). According to preference analysis, we found specific endophyte taxa consistently showing tissue preference regardless of the host species. For example, *M. ibericus* was found exclusively in roots from all ten desert halophytes in the current study. The taxon was firstly described from healthy roots of *Atriplex portulacoides*, *Plantago crassifolia*, and an undetermined plant in saline habitats of Spain (Collado et al. 2002). *Monosporascus* spp. are well known as pathogens infecting fruit in Cucurbitaceae and vine growing in hot semi-arid climates with soils that tend to be saline and alkaline (Collado et al. 2002). Some members of *Monosporascus* spp. have been reported as root endophytes with a much broader host range, i.e., *Acleisanthes lanceolatus*, *Bouteloua gracilis*, *Eustachys petraea*, *Mentzelia perennis*, *Nama carnosum*, *Nerisyrenia linearifolia*, *Sartwellia flaveriae*, and *Tiquilia hispidissima* from Mexico, Honduras, and New Mexico (Porras-Alfaro et al. 2008; Herrera et al. 2010; Herrera et al. 2013; Porras-Alfaro et al. 2014). Our study shows that *Al. eichhorniae* predominated the endophyte assemblages and preferred to colonize the stems rather than the roots. *Alternaria* fungi as dominant endophytes showing preference in specific tissues but very low specificity with respect to host species, were mainly isolated from leaves in six halophytes in inland salt marsh of Canada (Muhsin and Booth 1987), in eight halophytes in Tennger Desert of China (Sun et al. 2012b), and in eight gypsophilous flowering plants in New Mexico desert (Porras-Alfaro et al. 2014). These previous studies in halophytes and desert gypsophytes indicated that some endophytic fungi show strong tissue preferences.

## Conclusions

The present study revealed high diversity of endophytic fungi associated with desert halophytes, and their colonization rate and diversity of endophytic fungi vary from plant to plant and is higher in roots than in stems. The endophytic fungal community composition is affected by plant species and tissue type as some endophytic fungi showed strong host and tissue preferences. The current study will provide preliminary data for exploration into diverse bioactive natural products originated from halophyte endophytes, and prospects on ecosystem reconstruction or desert agriculture development.
